# The Indiscriminate Sale of Poisons

**Published:** 1872-06

**Authors:** 


### EDITORIAL AND MISCELLANEOUS.

### THE INDISCRIMINATE SALE OF POISONS.

The following editorial, of deep, general and professional interest, is extracted from the Boston *Medical and Surgical Journal:*

"CORONER'S VERDICT.—In the case of Lizzie Whittemore the jury of Coroner Hastings brought in the following verdict:

"That the said Lizzie Whittemore came to her death at eleven A.M., June 3, 1872, at 27 Kneeland Street, in consequence of excessive use of alcoholic liquors and an overdose of morphine, administered by some person or persons unknown to the jury; and they further find that the custom of selling deadly poisons by apothecaries (as in this instance) to persons unknown to them, and without prescriptions from physicians, is highly culpable."

Again, and for the hundredth time, a coroner's jury has had occasion to deliberate upon a mysteriously sudden death, whose cause it required only brief investigation and moderate acumen to determine. The story is a short and familiar one. A young girl, friendless and alone, in a great city, tired of life because some of its mad schemes have disappointed her, resolves to destroy herself. The means for the fulfillment of her purpose are manifold, but the adjacent apothecary is selected to provide the sure and speedy poison. For cash, the abundant supply of narcotic is readily obtained on demand, and no questions are asked. The way is now clear; the poison is taken, the coroner's jury holds its sessions and returns a verdict, and the case is forgotten. But the lesson it teaches should not be forgotten, but should be told again and again, with renewed emphasis. The closing clause in the verdict of Coroner Hasting's jury, in the case above alluded to, reiterates it in terms which are beyond cavil, and which will be approved by the judgment of all sensible people, in and out of the profession, druggists included. Such plain and forcible expressions, concerning a too common and too dangerous custom, deserve the highest commendation and approval.

There are laws whose letter and spirit are sufficiently explicit in their prohibition of the indiscriminate sale of poisonous drugs, and which regulate the barter in such articles by provisions and limitations abundantly calculated as safeguards; but hardly a day passes that the newspapers do not record deaths by suicide or by misadventure resulting from the careless or the malicious violation of these restrictive statutes. Yet, who has ever heard of a prosecution, under these acts, against a druggist?

Nor do the apothecaries themselves hesitate to acknowledge their own free interpretation of the law in their practice. In an investigation, undertaken the last year in this State by the Board of Health, to determine the relative increase in the opium-habit, the druggists of this city were canvassed; and out of a large number, only two or three declared that it was their rule never to dispense narcotics except by prescription.

We give it as a hint to coroners' juries, and as one method of meeting this growing evil, that when they are called upon to render such a verdict as that which heads this article, it will have a salutary effect if, instead of making the blame of general application, they fix the culpability on the offending druggist in particular. The number of suicides is not so great that a druggist so well-disposed to suicidal purposes would find the calls for poisons greatly increased, because of the exposure; while the effect on his fellow-pharmacists would have a good tendency.

But the apothecaries must not be made to take all the blame in this matter. Physicians are not altogether above reproach, and much of the looseness of poison-dispensing is fairly chargeable to them. It is a frequent habit with many practitioners, especially in their visits to families with whom they enjoy a certain degree of professional intimacy, to speak of drugs and to order them with a heedless freedom which deprives the poisonous articles of their deleterious character in the minds of the consumers. Especially is this the case with the preparations of opium. If a child is in pain, the general direction to "give it five or ten drops of paregoric" is deemed enough without a prescription. If an opiate fomentation is required, the advice is to "get a couple of ounces of laudanum at the druggist's" for the purpose. This heedlessness is easily acquired, but it is none the less blameworthy. It reacts on those who use the drugs, to throw them off their guard; and on those who sell them, to give them a ready excuse for similar heedlessness in dispensing. At best, physicians' prescriptions are, as a rule, sufficiently loose in their construction, and a regular course of practical instruction in this subordinate but important department of the medical art would be an acceptable innovation. But, meantime, for those to whom the medicamenta are familiar therapeutic instruments, in their daily practical duty, the lesson can not be learned too soon, that the written prescription, even kept in duplicate, is not labor lost, no matter how trivial the order; that every such prescription is a voucher for the physician, for the patient, and for the druggist; and that it is a pledge of accuracy which may greatly aid to correct the evils of indiscriminate dispensing.

